# Silencing of Testin expression is a frequent event in spontaneous lymphomas from *Trp53*-mutant mice

**DOI:** 10.1038/s41598-020-73229-3

**Published:** 2020-10-01

**Authors:** Robert J. Weeks, Jackie L. Ludgate, Gwenn Le Mée, Rubina Khanal, Sunali Mehta, Gail Williams, Tania L. Slatter, Antony W. Braithwaite, Ian M. Morison

**Affiliations:** Department of Pathology, Dunedin School of Medicine, University of Otago, Dunedin, New Zealand

**Keywords:** Cancer epigenetics, Tumour-suppressor proteins

## Abstract

The tumour suppressor gene, *TES*, is frequently methylated in many human tumours. Previously, we demonstrated that *TES* promoter methylation and transcriptional silencing was the most common molecular abnormality detected in childhood acute lymphoblastic leukaemia (ALL). *Trp53*-mutant mouse models predominantly develop B- and T-cell lymphomas, which are widely considered equivalent to childhood T and B ALL. In this study, we examined expression of *Tes* transcript and Testin protein in spontaneous tumours obtained from three *Trp53*-mutant mouse models. Using immunohistochemistry, we report that 47% of lymphomas lacked Testin protein compared to only 7% of non-lymphoid tumours. Further examination of the lymphomas from *Trp53-null* and *Trp53-mΔpro* homozygous mutant mice revealed that 63% and 69% respectively of the isolated lymphomas were Testin negative, which is similar to reported rates in childhood T-ALL. Surprisingly, lymphomas from *Trp53-Δ122* mice were frequently Testin positive (> 60%), suggesting that the presence of the Trp53-Δ122 protein appeared to mitigate the requirement for *Tes* silencing in lymphomagenesis. Quantitative RT-PCR results confirmed that this lack of Testin protein was due to *Tes* transcriptional silencing, although bisulfite sequencing demonstrated that this was not due to promoter methylation. These results are consistent with the Testin protein having lymphoid tumour suppressor activity in both mice and humans.

## Introduction

Silencing of the *TES* gene is emerging as a common event during tumourigenesis with loss of TESTIN protein observed in many tumours, including glioblastoma^1–3^, gastric^4^, uterine^5^, ovarian^6^, prostate^7^ and breast^5,8^. Loss of TESTIN protein is predominantly the result of promoter methylation, with mutations of the *TES* coding sequence rarely reported. Previously, we reported that *TES* promoter methylation was a common event across all subtypes of childhood acute lymphoblastic leukaemia (ALL) and that dense, biallelic methylation of the *TES* promoter results in loss of transcription and absence of TESTIN protein^9^. In addition, re-expression of TESTIN in human ALL cell lines and other cancer cell lines resulted in suppression of anti-apoptotic proteins and induction of pro-apoptotic proteins^10^, resulting in increased cell death^1,8,10–14^, thus adding support for *TES* to be considered a tumour suppressor gene.

The high prevalence and clonal nature of *TES* silencing in paediatric ALL strongly suggests that *TES* silencing is either an early event or an epigenetic driver of leukaemia development. In this study, we investigated the role of *TES* expression in spontaneous tumours isolated from tumour-prone, *Trp53*-mutant mice. TP53 is a tumour suppressor that regulates the expression of multiple target genes and thereby can induce cell cycle arrest, DNA repair, apoptosis, senescence, or changes in metabolism in response to cellular stress^15^. *TP53* gene mutations are common in human cancers and haematological neoplasms^16^. *Trp53*-mutant mice are susceptible to spontaneous T- or B-cell lymphomas development^17–19^, which are widely considered equivalent to childhood T and B ALL^20^. Of the three mouse models, the *Trp53*-null mice are unable to produce Trp53 protein and die rapidly, predominantly from T (56%) or B cell (18%) lymphomas^21^. The *Trp53mΔpro* mice produce a mutant Trp53 protein without the proline-rich domain (amino acids 58–88) and have a reduced lifespan compared to wild-type mice, succumbing to B-cell lymphomas (50%), osteosarcomas and T-cell lymphomas^18^. The third *Trp53*-mutant model produces the truncated Trp53-Δ122 protein, equivalent to the human Δ133p53 oncogenic isoform that has been observed in multiple tumours^19,22^. The Trp53-Δ122 protein promotes migration and actin polymerisation and *Trp53-Δ122* mutant mice have an inflammatory phenotype, and succumb rapidly to B and T cell lymphomas^18,23–25^. As these *Trp53-Δ122* mutant mice develop tumours more rapidly than *Trp53*-null mice, the Trp53-Δ122 protein is considered to be oncogenic.

## Results

### Loss of Testin in Trp53-mutant lymphomas

**Figure 1:**
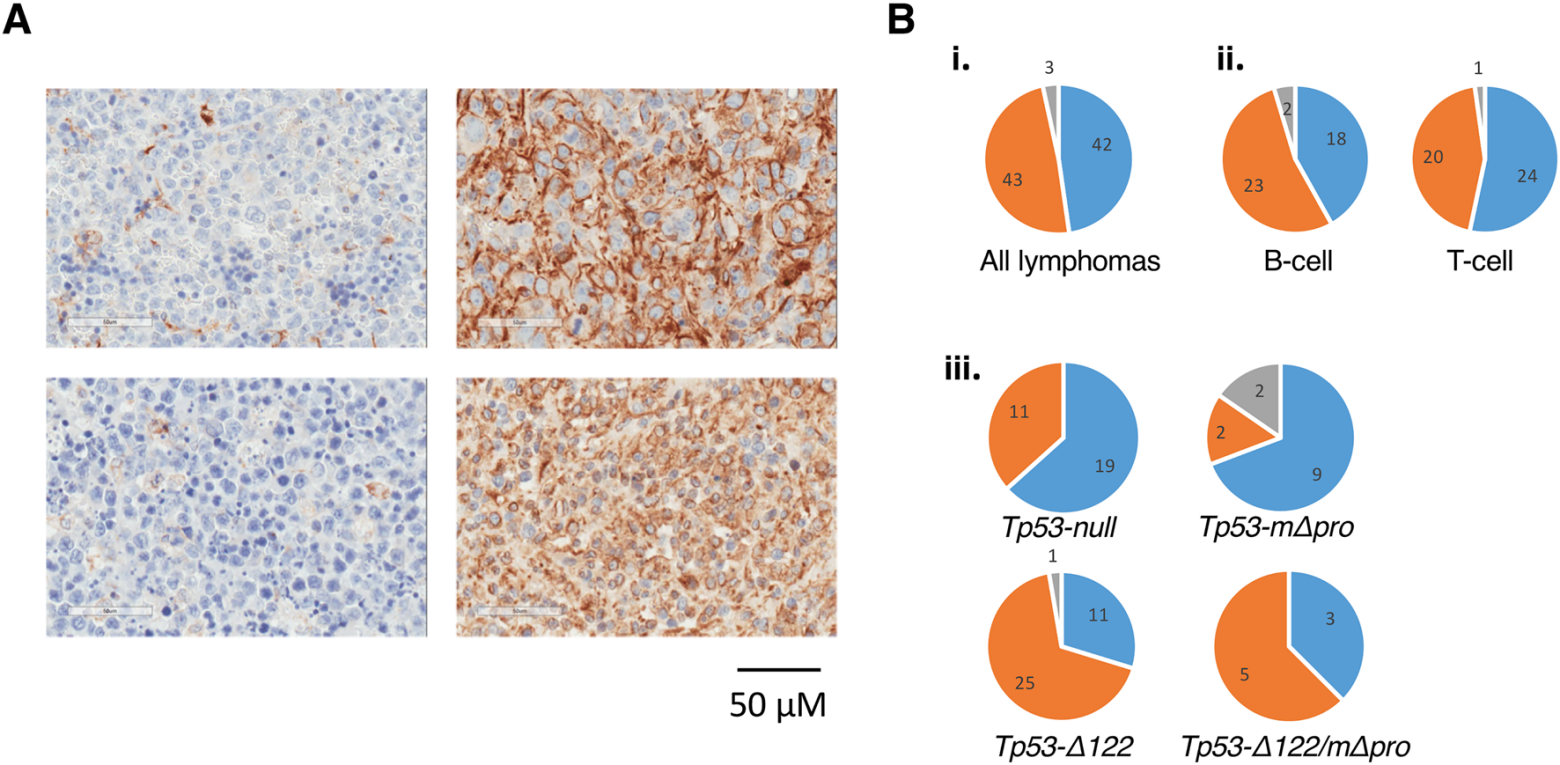
Testin immunohistochemistry of lymphomas. (**A**) Examples of immunohistochemistry results showing Testin-positive (right) and Testin-negative (left) lymphoma cells in tumours in the spleen (upper) and thymus (lower). (**B**) Pie-charts showing numbers of mice with lymphomas with respect to Testin status. (**i**) All lymphomas. (**ii**) B- or T-cell lymphomas. (**iii**) *Trp53*-mutant genotype of lymphomas. (Testin status: blue box negative; orange box positive; grey box mixed).

Tumours from *Trp53*-mutant mice were collected and characterised using haematoxylin and eosin staining and immunohistochemistry (IHC) with antibodies to B cell (B220) and T-cell (CD3) surface markers. In addition, both lymphoid and non-lymphoid tumours were classified according to Testin protein status by IHC (see Fig. 1A for examples). Overall, the non-lymphoid tumours [osteosarcomas (n = 3), rhabdomyosarcoma (n = 1), malignant fibrous histiocytomas (n = 9), hamartoma (n = 1) and one undefined], were Testin-positive with only one testin-negative tumor [1 of 15 (7%); data not shown] compared to 42 of 88 (48%) lymphomas being negative for Testin protein (Fisher’s exact test; P = 0.0018) (Supplementary Table S1).

Closer examination of the IHC results revealed some interesting observations (Fig. 1B). Firstly, a similar proportion of B- and T-cell lymphomas were Testin-negative (42% of B-cell and 53% of T-cell lymphomas), which is in contrast to childhood ALL, where 94% of B-ALL and 71% of T-ALL were TESTIN negative^9^.

Secondly, Testin positive lymphomas were not observed at similar frequencies within the *Trp53*-mutant mice groups. For example, lymphomas isolated from *Trp53-Δ122* mice were largely Testin positive or mixed (70%), whereas those from homozygous *Trp53-null* and homozygous *Trp53-mΔpro* mice were predominantly negative for Testin protein (63% and 69%, respectively).

Using Fisher’s exact test (2-tailed), the significance of the Testin status for each *Trp53*-mutant group with respect to the *Trp53-Δ122* allele was calculated (Table 1)*.* In brief, these results demonstrate that the *Trp53*-*Δ122* allele was associated with the presence of Testin protein in the lymphomas, despite some comparisons failing to reach significance (P < 0.05). For example, the proportion of Testin positive and negative lymphomas isolated from *Trp53*-*Δ122* was significantly different when compared to either homozygous *Trp53*-*null* (Group 1, P = 0.013) or homozygous *Trp53*-*mΔpro* (Group 2, P = 0.0044) mice, but not when compared to the small number of heterozygous*Trp53*-*mΔpro/Trp53*-*Δ122* mice (Group 3, P = 0.7).

**Table 1:** Contingency table for lymphomas with respect to *Trp53*-mutant genotype and Testin IHC status.

Groups	*Trp53* genotype	No. of lymphomas		Significance level
		Testin positive	Testin negative	
Group 1	Null/null	11	19	**P = 0.013**
	Δ122/Δ122	25	11	
Group 2	mΔpro/mΔpro	2	9	**P = 0.0044**
	Δ122/Δ122	25	11	
Group 3	Δ122/mΔpro	5	3	P = 0.70
	Δ122/Δ122	25	11	
Group 4	Null/null	11	19	P = 0.24
	Δ122/mΔpro	5	3	
Group 5	mΔpro/mΔpro	2	9	P = 0.074
	Δ122/mΔpro	5	3	
Group 6	Null/null	11	19	**P = 0.0021**
	Δ122 any	30	14	
Group 7	mΔpro/mΔpro	2	9	**P = 0.0010**
	Δ122 any	30	14	
Group 8	non-Δ122	13	28	**P = 0.0001**
	Δ122 any	30	14	

Significance was calculated using Fisher’s exact test (2-tailed).

Comparing the heterozygous *Trp53*-*mΔpro/Trp53*-*Δ122* mice with the homozygous *Trp53*-*null* (Group 4, P = 0.24) or with the *Trp53*-*mΔpro* mice (Group 5, P = 0.074), did not reveal significant differences in proportions of *Tes*-positive lymphomas, likely due to the low numbers of heterozygous mice present (n = 8)*.*

To further investigate the effect of the Δ122 protein on Testin status, we combined the homozygous *Trp53*-*Δ122* and heterozygous *Trp53*-*mΔpro/Trp53*-*Δ122* mice into the “Δ122-any” cohort. Comparison of the proportion of *the Tes*-positive lymphomas between this “Δ122-any” cohort and the homozygous *Trp53*-*null* mice (Group 6, P = 0.0021) and between the homozygous *Trp53*-*mΔpro* mice (Group 7, P = 0.001) were highly significant. And finally, comparison of this “Δ122-any” cohort with the combined “non-Δ122 mice” (homozygous *Trp53*-*null* mice and homozygous *Trp53*-*mΔpro*) (Group 8, P = 0.0001) revealed highly significant differences in the proportions of Testin-positive lymphomas.

Firstly, these results confirm that silencing of *Tes* is frequently observed in lymphoid tumours and is likely required for lymphoma development. And secondly, that the presence of the *Trp53*-*Δ122* allele increased the probability of a lymphoma being Testin-positive, suggesting that the presence of Trp53-Δ122 oncogenic protein reduced the requirement for Testin silencing in lymphoid tumour development.

As Testin silencing was frequently observed in *Trp53*-mutant lymphomas, we speculated that the presence of Testin protein would be protective for lymphoma onset and mouse survival. However, no statistically significant difference in survival for the Testin negative or positive lymphomas was observed within the genotype groups or between the CD3+ and B220+ lymphomas, although the numbers of mice within these comparisons were small (Supplementary Table S2).

### Testin protein is regulated by transcriptional control

**Figure 2:**
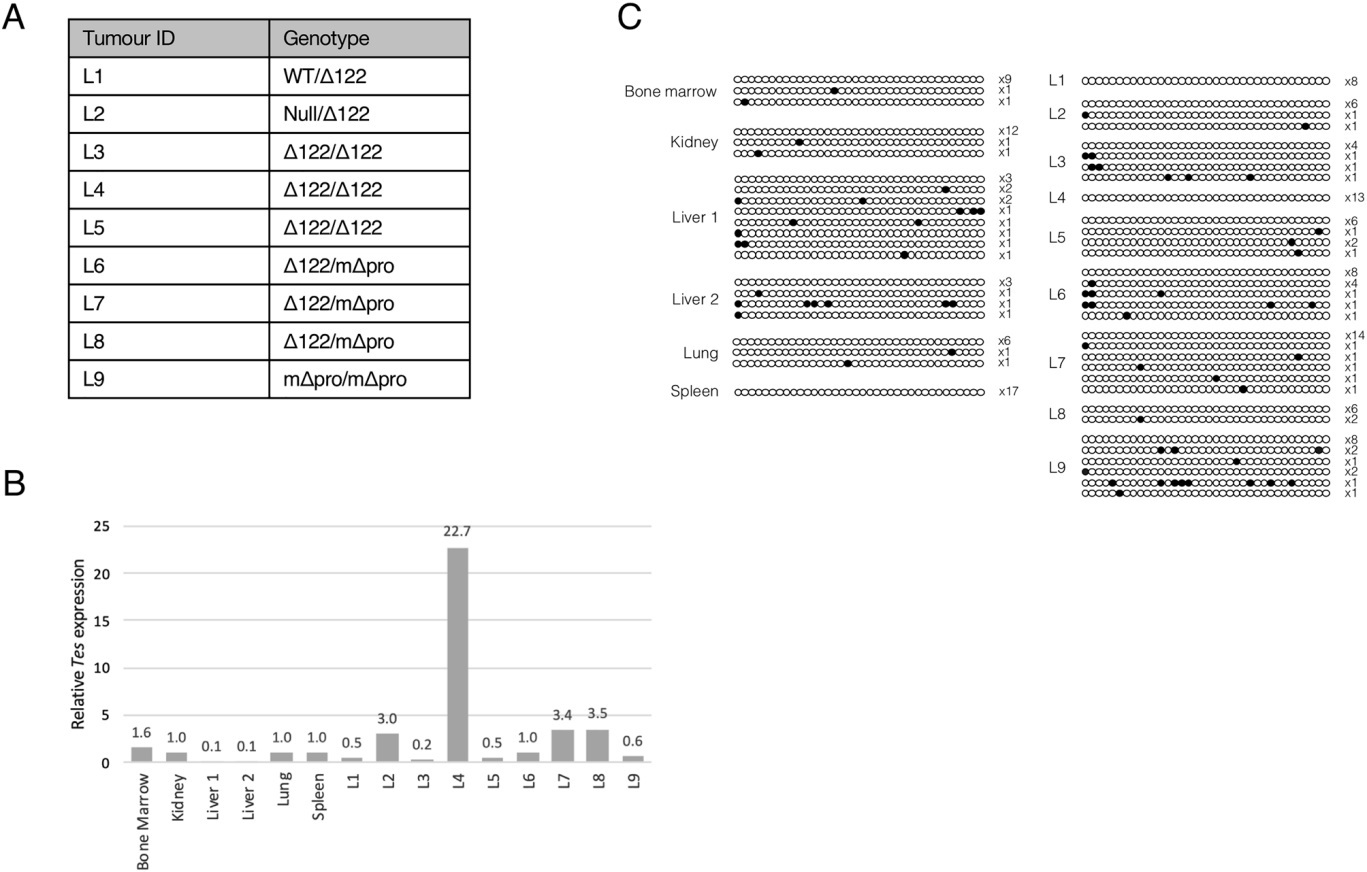
*Tes* expression and promoter methylation in *Trp53*-mutant lymphomas. (**A**) Table showing the genotype for the *Trp53*-mutant lymphomas tested. (**B**) Quantitative RT-PCR results showing *Tes* mRNA expression levels calculated relative to *Rps29* and *β2m* expression and normalised to spleen expression level for lymphomas and normal mouse tissues. Relative *Tes* expression levels are shown. (**C**) Methylation lollipop plots for normal tissues and lymphomas showing only the unique methylation patterns obtained. The observed frequency for each unique clone pattern is shown.

To determine whether the absence of Testin protein in mouse lymphomas was due to transcriptional silencing as is observed in childhood ALL, *Tes* transcript levels were quantified in a separate cohort of lymphomas and normal control tissues (Fig. 2A for details). Small pieces (1 mm^3^) of lymphoma, containing both normal and tumour cell populations of unknown proportions, were used for nucleic acid purification. Total RNA from these small lymphoma samples were used to determine *Tes* mRNA expression and quantitative RT-PCR results demonstrated that *Tes* transcript was reduced in 4 of the 9 lymphomas tested (Fig. 2B). As normal cells express *Tes* transcript (Fig. 2B), these results confirm that mouse *Tes* RNA expression is reduced or absent in lymphomas.

### Testin protein is not regulated by Tes promoter methylation in murine lymphomas

In childhood ALL, RNA transcriptional silencing and lack of TESTIN protein results from dense, biallelic *TES* promoter methylation^9^. To investigate whether *Tes* expression is similarly controlled by promoter methylation in the mouse lymphomas, we designed bisulfite-specific primers to amplify the CpG island located in the orthologous region of the mouse promoter.

As lymphoma samples contained both normal and tumour cells, we investigated promoter methylation using clonal bisulfite sequencing (see “Methods”). From bisulfite sequencing analysis (Fig. 2C), mouse lymphomas were largely unmethylated at the *Tes* promoter, although low-level, sporadic methylation was observed. It is improbable that this observed low level of methylation (< 5%), which was neither clonal, dense or present at specific CpG sites, is able to silence *Tes* transcription in mouse lymphomas. Therefore, we conclude that the observed reduction in *Tes* transcription in these lymphomas is not mediated via promoter methylation.

## Discussion

Promoter methylation and subsequent *TES* transcriptional silencing is observed in all molecular sub-types of childhood ALL^9^. TESTIN has been shown to inhibit cellular proliferation and increase apoptosis^11^ in ALL cells and other cancer cell lines^1,8,10–14^, confirming that *TES* is a tumour suppressor gene. The high prevalence and clonal nature of *TES* silencing in paediatric ALL strongly suggests that *TES* silencing is either an early event or an epigenetic driver of leukaemia development. To confirm the importance of *TES* transcriptional silencing in leukaemogenesis, we investigated *Tes* RNA and Testin protein expression in archived tumours isolated from tumour-prone mice.

In common with the prevalence of *TES* silencing observed in childhood ALL, we demonstrated that *Tes*-silencing and lack of Testin protein was common in spontaneous lymphomas isolated from *Trp53*-mutant mice, but was rare in non-lymphoid tumours. For the *Trp53-null* and *Trp53-mΔpro* homozygous mutant mice, 63% and 69% respectively of the isolated lymphomas were Testin negative, which is similar to reported rates in childhood T-ALL^9^. These results are consistent with Testin protein being a lymphoid tumour suppressor in both mice and humans.

Of surprise was the observation that lymphomas from mice expressing Trp53-Δ122 were frequently Testin positive (> 60%), suggesting that the presence of the Trp53-Δ122 protein appeared to mitigate the requirement for *Tes* silencing in lymphomagenesis. Trp53-Δ122 protein is considered to be oncogenic, as *Trp53-Δ122* mice demonstrate increased cellular proliferation, increased inflammation and die earlier with more aggressive tumours than null mice^19^. Following these surprising results, we propose that Trp53 and Testin have complementary functions in lymphoid tumour development and that this may be mediated via interacting proteins. Two recent reports have identified proteins which interact with TESTIN and are able to modulate TP53 activity. Firstly, ELL2 is a binding partner for TESTIN^26^ and ELL proteins can bind to the transactivation domain of TP53 and inhibit transactivation activity^27^. Secondly, Zyxin, a known binding partner of TESTIN^28^, has been reported to modulate the TP53/HIPK2 pathway by stabilising the HIPK2 protein, which phosphorylates Ser46 of TP53 and induces apoptosis^29^. These observations confirm that the TESTIN protein may modulate TP53 activity via protein interactions. However, as the Trp53-Δ122 protein lacks both Ser46 and the transactivation domain, these reported interactions are unable to explain our results with the *Trp53-Δ122* mice.

The lack of Testin silencing in tissues from the *Trp53-Δ122* mice suggests that Trp53-Δ122 protein can override the mechanism of silencing. A possible explanation for this comes from a report that the human Δ133p53 isoform can overcome cell growth arrest induced by p53 family members, p63 and p73^30^. Thus, p63 and/or p73 could repress Testin expression, but when Δ133p53 is over-expressed, this repression is prevented. Preliminary data consistent with this explanation are shown in Supplementary Fig. S1. In three separate cohorts of haematopoietic tumors, *p73* (but not *p63* or *p53*) mRNA expression was negatively correlated with *TES* expression. There is also a p53/p63/p73 response element within the *TES* promoter^31^.

Previously, we demonstrated that silencing of *TES* transcription by promoter methylation is prevalent in childhood ALL^9^ and that re-expression of TESTIN in human ALL cell lines and other cancer cell lines resulted in increased cell death^11^, thus adding support for *TES* to be considered a tumour suppressor gene. In this study, we show that silencing of *Tes* transcription and subsequent lack of Testin protein is common in equivalent mouse tumours, supporting its proposed role in suppressing lymphoid tumour development.

## Methods

### Mouse husbandry and genotyping PCR

As homozygous *Tp53*-mutant mice were reported to have fertility problems^32^, all breeding crosses were performed with heterozygous *Tp53*-mutant mice. All mice were tail-tipped at weaning and tails were digested with proteinase K overnight. *Tp53*-specific PCR amplifications of crude tail-tip preparations were used to identify *Tp53* genotypes (details available on request). Mutant mice were maintained under standard housing conditions and monitored for tumour development, such as morbidity, swelling of the abdomen and hunching. Mice were euthanised once tumours or morbidity were detected and tumors and normal tissues were collected and fixed in 10% neutral-buffered formalin or RNA*later* (Life Technologies Ltd).

Mouse studies were approved and conducted in accordance with local guidelines and regulations under University of Otago Ethics Approvals—AEC 20/07, 21/07 and D118/09.

### Immunotyping of lymphomas

Tissues and tumours were paraffin-embedded and sections were stained with haematoxylin and eosin. Tumours were classified by a mouse pathologist (GW) and T- and B-cell lymphomas were identified after immunohistochemistry with CD3 and B220 antibodies, respectively. Testin antibody (SC-100914 TES (AA-7), Global Science) was optimised on mouse spleen and human tonsil tissue sections (see Supplementary Fig. S2) and validated using tissues isolated from homozygous *Tes*-genetrap mice^33^ (data not shown). The Testin status of the tumours isolated from *Tp53*-mutant mice was determined using Testin antibody and immunohistochemistry, using the following scoring system: lymphomas with fewer than 10% of tumour cells positive for Testin antibody staining were classified as ‘negative’; whereas lymphomas with more than 80% of their tumour cells being Testin positive were classified as ‘positive’. Furthermore, the small number of lymphomas with less than 80%, but more than 20% of cells being positive for Testin antibody staining were labelled as ‘mixed’.

### Quantitative RT-PCR for Tes transcript

A small, separate cohort of lymphomas (Supplementary Table S1) containing both normal and tumour cells were collected into RNA*later* and stored at − 20 °C. Genomic DNA and total RNA was isolated (*MN RNA Isolation kit*) from small biopsies (1 mm^3^) of these lymphomas and from fresh, normal tissues. RNA was reverse-transcribed to cDNA using the *High Capacity cDNA Reverse Transcription kit* (ThermoFisher Ltd). Quantitative RT-PCR was performed in triplicate for *Tes* (IDT PrimeTime assay-Mm.Pt.56a.43509122 (exons 6–7); Integrated DNA Technologies), *β2-microglobin* (IDT PrimeTime assay-N009735.1.pt; Integrated DNA Technologies) and *Rps29* (TaqMan assay-Mm02342448_gH; Applied Biosystems Ltd)) using Takara 2 × qPCR mix and a LightCycler real-time PCR machine. *Tes* expression was quantified using the ΔΔCt calculation method with *Rps29* and *β2M* expression as reference genes and normalised to mouse spleen expression levels.

### Bisulfite sequencing of the murine Tes promoter

In order to investigate DNA methylation at the mouse *Tes* promoter, we designed bisulfite-specific primers to the orthologous region previously investigated in the human gene^9^. Genomic DNA from lymphomas and normal tissues were bisulfite-treated using *EZ DNA Methylation Gold Kit* (Zymo Research Ltd). *Tes* promoter regions were amplified with bisulfite-specific primers (forward: GGG TTA TTT ATT TTT TTT GGT TTG TT; reverse: TTT AAT TTC CAA ATC CAT ACT AAA C; product is 451 bp in length) and *KAPA HiFi Hotstart Ready Mix*, using the following program (98 °C for 45 s; 98 °C for 15 s, 56.7 °C for 30 s, 72 °C for 30 s, for 35 cycles; 72 °C for 5 min). PCR products were cloned using the TOPO Cloning kit (Life Technologies Ltd) and DH5α competent cells. Plasmid DNA was prepared (*Zyppy Plasmid miniprep kit*, Zymo Research Ltd) from bacterial colonies and sequenced according to established protocols.

## Supplementary information

Supplementary Information 1

Supplementary Figure 1

Supplementary Figure 2

Supplementary Table Legends

## Data Availability

The datasets used and/or analysed during the current study are available from the corresponding author on reasonable request.

## Abbreviations

IHC: Immunohistochemistry
RT-PCR: Reverse-transcriptase polymerase chain reaction
ALL: Acute lymphoblastic leukaemia
