# Supplementary material for: Silencing of Testin expression is a frequent event in spontaneous lymphomas from Trp53-mutant mice
Source: Sci Rep. 2020 Oct 1;10:16255. doi: 10.1038/s41598-020-73229-3 (PMC7530732; doi:10.1038/s41598-020-73229-3)
Supplement: Supplementary file 2 — Supplementary Figure 1 [file 41598_2020_73229_MOESM2_ESM.docx]

**Silencing of Testin expression is a frequent event in spontaneous lymphomas from *Trp53*-mutant mice**

Robert J. Weeks*, Jackie L. Ludgate, Gwenn Le Mée, Rubina Khanal, Sunali Mehta, Gail Williams, Tania L. Slatter, Antony W. Braithwaite, Ian M. Morison

*Address: Department of Pathology, Dunedin School of Medicine, University of Otago, New Zealand.*

**Corresponding author:* [*rob.weeks@otago.ac.nz*](mailto:rob.weeks@otago.ac.nz) *(*https://orcid.org/0000-0003-0474-9089)


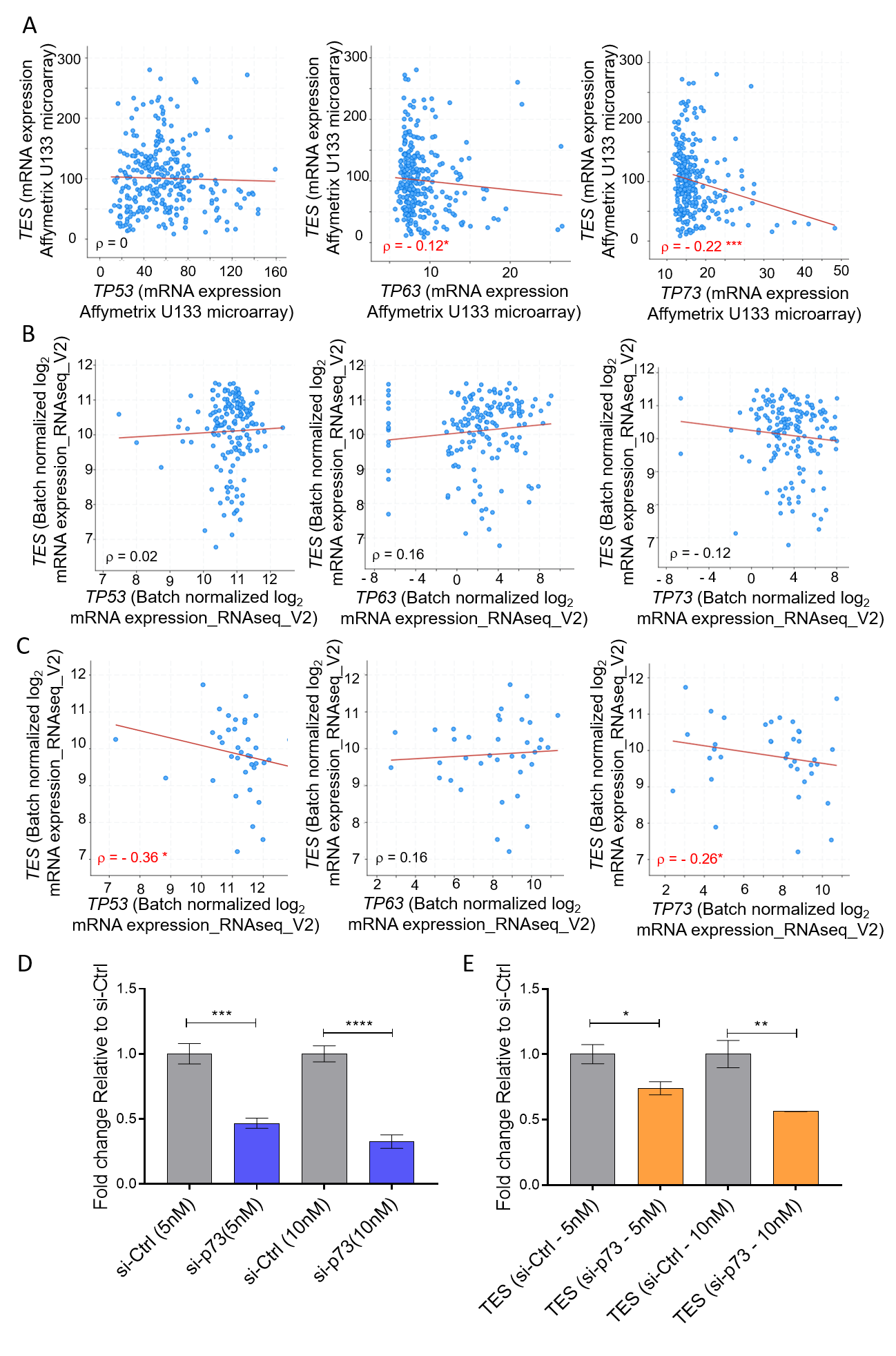


**Legend to figure**

**Supplementary Figure S1: *TP73* is negatively associated with *TES* mRNA expression. A-C.** Data was obtained from cBioPortal (1). Association of *TP53* (left panel), *TP63* (middle panel) and *TP73* (right panel) mRNA with *TES* mRNA expression. Each dot represents a tumour sample and the red line - line of best fit. ρ = Spearman’s correlation coefficient. Significance was determined by two-tailed t-test. * - p < 0.05, *** - p < 0.001.  **A.** Mature B-cell (M.D. Anderson dataset; n = 290), **B.** Acute Myeloid Leukemia (PanCancer dataset; n = 200) and **C.** Diffuse B-Cell lymphoma (PanCancer dataset; n=37).
