# Supplementary material for: Silencing of Testin expression is a frequent event in spontaneous lymphomas from Trp53-mutant mice
Source: Sci Rep. 2020 Oct 1;10:16255. doi: 10.1038/s41598-020-73229-3 (PMC7530732; doi:10.1038/s41598-020-73229-3)
Supplement: Supplementary file 3 — Supplementary Figure 2 [file 41598_2020_73229_MOESM3_ESM.docx]

**Silencing of Testin expression is a frequent event in spontaneous lymphomas from *Trp53*-mutant mice**

Robert J. Weeks*, Jackie L. Ludgate, Gwenn Le Mée, Rubina Khanal, Sunali Mehta, Gail Williams, Tania L. Slatter, Antony W. Braithwaite, Ian M. Morison

*Address: Department of Pathology, Dunedin School of Medicine, University of Otago, New Zealand.*

**Corresponding author:* [*rob.weeks@otago.ac.nz*](mailto:rob.weeks@otago.ac.nz) *(*https://orcid.org/0000-0003-0474-9089)

**
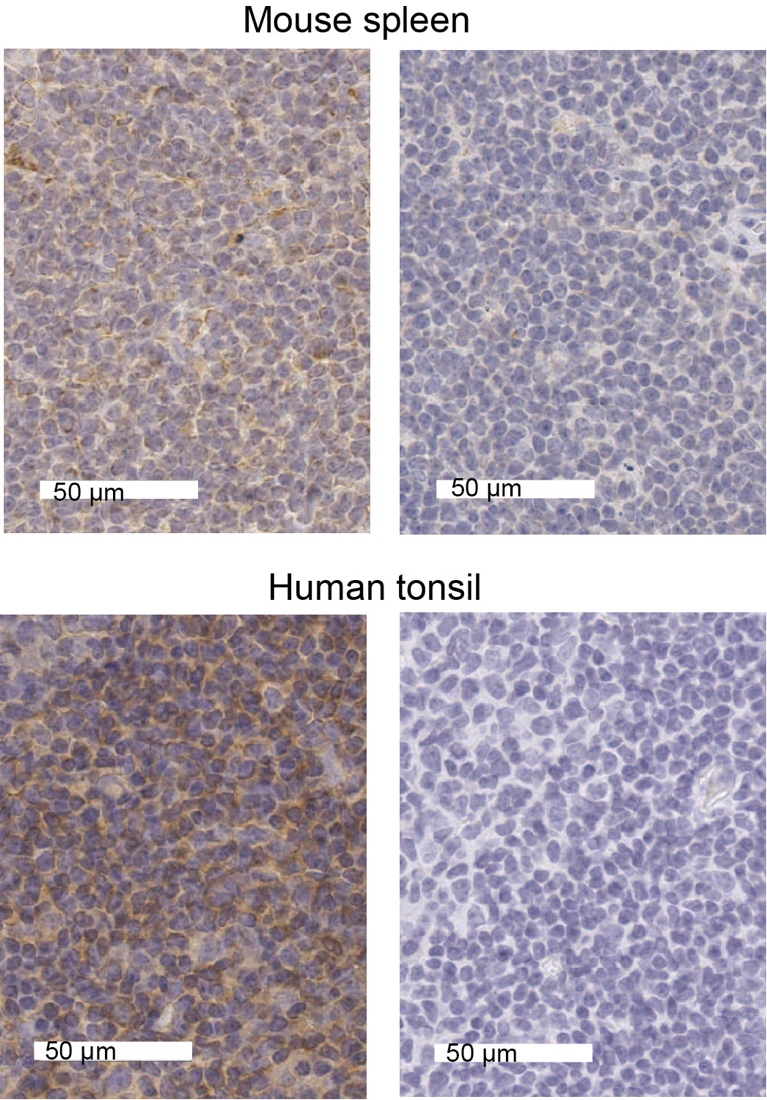
**

**Legend to figure**

**Supplementary Figure S2. IHC images showing Testin antibody staining of control tissues.** IHC images of mouse spleen sections stained with Testin antibody (top left), and the no primary antibody control (top right) and lower panels show human tonsil with Testin antibody (bottom left) and the no primary antibody control (bottom right).
