# Supplementary material for: Silencing of Testin expression is a frequent event in spontaneous lymphomas from Trp53-mutant mice
Source: Sci Rep. 2020 Oct 1;10:16255. doi: 10.1038/s41598-020-73229-3 (PMC7530732; doi:10.1038/s41598-020-73229-3)
Supplement: Supplementary file 4 — Supplementary Table Legends [file 41598_2020_73229_MOESM4_ESM.docx]

**Supplementary Table S1. Summary table showing IHC status of spontaneous lymphomas isolated from *Trp53-*mutant mice and the ages at which mice were euthanised.**

**Supplementary Table S2. Median age at death of the *Trp53*-mutant mice.** Summary table showing the median ‘age-at-death’ for the *Trp53-*mutant mouse, with respect to Testin status and for B220+ and CD3+ status.
